# Adipose Tissue-Derived Exosome and miR-142a-3p Alleviate Acute Lung Injury by Inhibiting HMGB1-Driven Autophagy

**DOI:** 10.3390/cells15030264

**Published:** 2026-01-30

**Authors:** Qianlin Long, Kejie Chen, Yizhu Li, Ruinan Peng, Yijian Yan, Jintao Ma, Jia Wang, Qiuyu Song, Yu Xue, Fengyuan Wang

**Affiliations:** 1 College of Animal & Veterinary Sciences, Southwest Minzu University, Chengdu 610041, China; 19180950867@163.com (Q.L.); 17360660996@163.com (Y.L.); 15380923397@163.com (R.P.); 18328373567@163.com (Y.Y.); 2 School of Public Health, Chengdu Medical College, Chengdu 610500, China; kejiechen930@cmc.edu.cn (K.C.); 18190982169@163.com (J.M.); 15208191387@163.com (J.W.); 18108271197@163.com (Q.S.); xy661213670710@163.com (Y.X.)

**Keywords:** acute lung injury, exosome, miR-142a-3p, autophagy

## Abstract

**Highlights:**

**What are the main findings?**

- Adipose-derived exosomes and miR-142a-3p attenuate LPS-induced ALI by suppressing HMGB1-driven autophagy in ALI.
- Exosomes from lean or DIO mouse exert comparable anti-autophagy effects and protect against LPS-induced ALI.

**What are the implications of the main findings?**

- Inhibition of pulmonary autophagy by regulating HMGB1 may contribute to a therapeutic effect.
- Exosomes and miRNAs targeting HMGB1 may hold potential as novel treatment strategies for ALI.

**Abstract:**

Acute lung injury (ALI) is a clinically severe respiratory disorder, of which autophagy is the crucial mechanism. Exosomes have the potential to treat ALI, but the role of adipose-derived exosomes (ADEs) in the autophagy of ALI remains unclear. Using an LPS-induced ALI model, the effects of ADE isolated from a lean or diet-induced-obese (DIO) mouse and ADE-carried miRNAs were investigated. After administration of ADEs, the levels of autophagy-related molecules were determined by qRT-PCR, Western blotting, and immunohistochemical staining. Then, a miRNA targeting *HMGB1* was screened by bioinformatic analysis and a dual-luciferase reporter assay, and its effect on the HMGB1-driven autophagy in an ALI mouse was investigated as ADEs. The data showed that LPS caused lung injury and activated HMGB1-driven autophagy. The ADEs from a lean mouse or DIO mouse significantly alleviated histopathological lesions, and they inhibited HMGB1-driven autophagy by down-regulating LC3, Beclin-1, and Atg5; the effects of ADEs were not significantly different between a lean and DIO mouse. Of the miRNAs carried by ADE, moreover, miR-142a-3p could specifically bind to *HMGB1* mRNA, and up-regulation of pulmonary miR-142a-3p suppressed HMGB1-driven autophagy and relieved lung injuries. Our results indicated that miR-142a-3p and ADEs mitigate LPS-induced ALI by inhibiting HMGB1-driven autophagy, providing new insights on the prevention and treatment of ALI.

## 1. Introduction

Acute lung injury (ALI) and acute respiratory distress syndrome (ARDS) are characterized by the release of inflammatory cytokines and immune cells triggered by extrapulmonary or pulmonary insults [1], manifesting with diffuse pulmonary interstitial and alveolar epithelial cell damage, non-cardiogenic pulmonary edema, and refractory hypoxemia. Presently, the primary treatment for ALI/ARDS is mainly symptomatic therapies such as mechanical ventilation and fluid management, but these treatments can only relieve ALI/ARDS symptoms, rather than improve the prognosis [2]. Consequently, the fatality rate of ALI patients remains high, up to 50% [3], and it is urgent to explore safe and effective therapeutic strategies or treatments for ALI and ARDS.

The exosome is a type of extracellular vesicle with a diameter of about 30~150 nm, and many cell types, such as stem cells, immune cells, and tumor cells, are capable of releasing exosomes [4,5,6]. Once formed, exosomes convey a variety of cargos, including miRNAs, proteins, and lipids, to recipient cells, and they can exert biological effects in cell communication and diseases [7]. It has been reported that exosomes derived from different cells can alleviate acute lung injury (ALI) by inhibiting alveolar macrophage pyroptosis [8,9], pulmonary inflammation [10,11], endothelial barrier dysfunction [11,12], and neutrophil extracellular trap formation [13]. These protective effects of exosomes could be attributed to the miRNAs carried by exosomes in ALI [14,15], and thus engineered or artificial exosomes loaded with miRNAs could serve as potential therapeutic approaches in the future [16].

Autophagy is a highly conserved and important process of intracellular material turnover in eukaryotic evolution, and it plays a multifaceted role in cellular homeostasis and pathological conditions, like infection, cancer, and Alzheimer’s disease [17]. In ALI, the effect of autophagy is still controversial. Although many researchers have reported that autophagy can protect against ALI by reducing organelle damage, inflammation, and oxidative stress [18], excessive activation of autophagy may lead to exacerbation of ALI [19,20,21,22]. And autophagy has been a pivotal target to explore potential therapeutic strategies for ALI.

High-mobility group protein B1 (HMGB1), widely distributed in the lungs, brain, liver, heart, and kidneys, is involved in replication, recombination, transcription, and DNA repair processes, and it has been shown to play an important role in autophagy regulation [23,24]. Previous studies showed that the increased levels of HMGB1 are correlated with severe lung injury and ARDS [25,26], and inhibited expression of HMGB1 improved survival of ALI/ARDS patients [27]. In the present study, focusing on autophagy and HMGB1, the potentials and mechanisms of adipose-derived exosome alleviating ALI were investigated using a mouse model, which was undertaken to provide new insights into exosomes’ use to treat ALI/ARDS.

## 2. Materials and Methods

### 2.1. Establishment of Diet-Induced-Obese (DIO) Mouse Model

Sixty six-week-old male ICR mice (25~30 g) were purchased from Chengdu Dashuo Laboratory Animal Co., Ltd. (Chengdu, China), and all animals were kept in the pathogen-free animal experimental center for 7 days before the experiment. Thereafter, all mice were randomly assigned to two groups: lean group (thirty mice) and DIO group (fifty mice). The mice in the lean group were fed with a conventional maintenance diet (1010001, Jiangsu Xietong, Nanjing, China), and the mice in the DIO group were fed with a 60% high-fat diet (XT19001, Jiangsu Xietong, Nanjing, China). After 12 weeks, the weights of mice were determined, and the mice in the DIO group with a 20% higher weight than those in the lean group were used as the DIO mice. Peripheral blood of DIO mice and lean mice was collected retro-orbitally, and sera of mice were separated through centrifugation and stored at −80 °C for further detection. Contents of serum total cholesterol (TC) and triglyceride (TG) were detected using commercial kits (A111-1 and A110-1, Nanjing Jiancheng Bioengineering Institute, Nanjing, China) as introductions. Levels of serum adiponectin and leptin were determined by ELISA kits (SEKM-0142, 4A biotech, Suzhou, China; CME0061, Solarbio life sciences, Beijing, China). Lee’s index of DIO mice and lean mice was calculated by the following formula:

$$\mathrm{Lee}’s\;\mathrm{index}\;=\;\frac{\sqrt[{3}]{\mathrm{Weight}\;(g)\;}\;\times \;1000}{\mathrm{Length}\;(\mathrm{cm})}$$

### 2.2. Extraction and Identification of Adipose-Derived Exosome

The DIO mice and lean mice were sacrificed by cervical dislocation, and then the abdomens of the mice were lightly sprayed with 75% alcohol until the abdominal hair was moist. A V-shaped incision was made from the abdomen to open the abdominal cavity, and all fat around the epididymis was collected and washed with pre-chilled PBS 2–3 times at 4 °C. Then, the adipose tissue was cut into small pieces and transferred into a six-well plate filled with 3 mL of complete DMEM, and it was put in an incubator (37 °C, 5% CO_2_). After 48 h, the medium supernatant was collected with a 1.5 mL EP tube, and the exosomes were extracted by differential centrifugation, where the specific method was as follows: the collected medium supernatant was centrifuged at 4 °C, 300× *g* for 10 min, and the pellet was discarded; we centrifuged at 4 °C, 2000× *g* for 10 min, then discarded the pellet to remove the cells; we centrifuged at 4 °C, 10,000× *g* for 30 min, then discarded the pellet to remove subcellular components; the supernatant in the EP tube was transferred to the ultracentrifuge tube; and then the supernatant was centrifuged at 100,000× *g* for 70 min, the precipitate was resuspended in pre-cooled PBS, and the exosomes were washed by centrifugation at 100,000× *g* for 70 min again. The supernatant was discarded, pellets were resuspended with 1 mL of pre-cooled PBS, and they were stored at −80 °C. The characteristics of exosomes from DIO mice (DIO-Exo) and lean mice (Lean-Exo) were detected with Western blotting, nanoparticle tracking analysis (NTA), and transmission electron microscopy (TEM).

### 2.3. Effect of Adipose-Derived Exosomes in the ALI Mouse Model Induced by LPS

Thirty-two six-week-old male ICR mice (25~30 g) were purchased from Chengdu Dashuo Laboratory Animal Co., Ltd. (Chengdu, China). All the animals were kept in the specific pathogen-free animal experimental center for 7 days before the experiment. Then, the mice were randomly divided into 4 groups (eight mice in each group), namely Con group, LPS group, LPS + D-Exo group, and LPS + L-Exo group. Con group: the mice were injected with sterilized saline through the tail vein, and intranasally instilled with sterilized saline after 1 h. LPS group: the mice were injected with sterilized saline through the tail vein, and intranasally instilled with LPS (5 mg/kg) after 1 h. LPS + D-Exo group: the mice were injected with DIO-Exo (100 μg/mL, in a total volume of 200 μL of phosphate-buffered saline) via the tail vein, and intranasally instilled with LPS (5 mg/kg) after 1 h. LPS + L-Exo group: the mice were injected with Lean-Exo (100 μg/mL) via the tail vein, and intranasally instilled with LPS (5 mg/kg) after 1 h. Following the determination of the total protein concentration of adipose-derived exosomes via the BCA method, a concentration of 100 μg/mL was set in accordance with the reported injection volumes of exosomes [28]. The concentration of LPS refers to our previous report [10]. The mice were weighed and sacrificed at 12 h post-treatment, and the lungs were weighed after collection. The lung coefficient was calculated as the following formula:

$$\mathrm{Lung}\;\mathrm{coefficient}\;=\;\frac{\mathrm{Lung}\;\mathrm{weight}\;(g)}{\mathrm{Body}\;\mathrm{weight}\;(g)}\;\times \;100\%$$

### 2.4. Prediction and Identification of miRNAs Targeting HMGB1 in the Lungs of ALI Mouse Model

#### 2.4.1. Prediction and Identification of miRNAs

miRNAs targeting HMGB1 were predicted through TargetScan 8.0 (https://www.targetscan.org (accessed on 25 October 2024)), microT-CDS of DIANA tools (http://diana.imis.athena-innovation.gr/DianaTools/ (accessed on 25 October 2024)), and RNAInter v4.0 (http://www.rnainter.org/ (accessed on 25 October 2024)) using default settings. Then, the miRNAs predicted by all 3 software were analyzed by Venn.

The levels of miRNA screened by bioinformatics analysis and mRNA in the lung were detected by quantitative real-time PCR (details in 2.8), and a correlation analysis of miRNA and mRNA in the lung was performed (details in 2.12).

#### 2.4.2. Dual-Luciferase Reporter Assay

The fragment of *HMGB1* 3′UTR, including the wild-type or mutant binding sites with miR-142a-3p, was sub-cloned into pmirGLO (Promega, Madison, WI, USA). The constructed reporters were termed as *HMGB1* 3′UTR-wt/mut. HEK-293 T cells were co-transfected with miR-142a-3p or miR-NC and reporter plasmids. The luciferase intensities were determined using the dual-luciferase reporter assay kit (E1910, Promega). Firefly luciferase intensity was normalized to Renilla luciferase activity. A dual-luciferase reporter assay was conducted three times, with three biological repetitions each time.

### 2.5. Effect of miR-142a-3p in the ALI Mouse Model

Twenty-four male ICR mice (25~30 g) were purchased from Chengdu Dashuo Laboratory Animal Co., Ltd. (Chengdu, China). All the animals were kept in the pathogen-free animal experimental center for 7 days before the experiment. Then, the mice were randomly divided into 3 groups (eight mice each group), namely the saline group, LPS group, and miRNA group. Saline group: the mice were injected with sterilized saline through the tail vein and intranasally instilled with sterilized saline after 1 h (note: the treatment of the saline group was same as the control group). LPS group: the mice were injected with sterilized saline through tail vein and intranasally instilled with LPS (5 mg/kg) after 1 h. miRNA group: the mice were injected with 5 nmol miR-142a-3p enhancer (agomir, miR4CM001, RiboBio Co., Ltd. Guangzhou, China) via the tail vein and intranasally instilled with LPS (5 mg/kg) after 1 h. The mice were weighed and sacrificed at 12 h post-treatment, and the lungs were weighed. The lung coefficient was calculated. Then, the lungs of four mice each group were collected and fixed in 4% paraformaldehyde, while the other lungs were harvested and stored at −80 °C for further detection. This study was carried out in strict accordance with guidelines developed by the China Council on Animal Care and Protocols. The protocol was approved by the Animal Ethics Committee (AEC) of the College of Animal & Veterinary Sciences, Southwest Minzu University (Approval Number: 202501222; Approval Date: 22 January 2025).

### 2.6. Lung Injury Assayed by Histopathology

After fixed with paraformaldehyde, the left lung of each mouse was embedded in paraffin and cut into 5 μm sections. Then, the tissue sections were dewaxed and processed with hematoxylin and eosin staining. Histopathological changes were observed and photographed with a digital camera (BX46F, Olympus, Tokyo, Janpan). A histological lesion of the lung was evaluated through ① the degree of hyperemia and bleeding; ② the degree of alveolar wall thickening; ③ inflammatory cell infiltration; and ④ exudate, and scored with 0–4 points according to the severity and invasion range of the above lesions.

### 2.7. Immunohistochemistry

The dewaxed sections of lungs were treated by 3.0% hydrogen peroxide blocking, boiling sodium citrate solution retrieval, 5% BSA blocking, and primary antibody (anti-HMGB1, anti-Beclin-1, and anti-Atg5 incubating overnight at 4 °C, respectively). Then, the sections were developed with SABC kits using protocols provided by the manufacturer (Boster Bio-engineering Limited Company, Wuhan, China, SA1020). The positive proteins were finally visualized by DAB and photographed with a digital camera (BX46F, Olympus, Tokyo, Janpan). The Integrated Optical Density (IOD) of positive protein was measured and analyzed using image-Pro Plus 6.0 (Rockville, MD, USA).

### 2.8. Quantitative Real-Time PCR

The collected mouse lung tissue is placed in trizol for grinding, and then total RNA is extracted and quantified to 1 μg. Then, the cDNAs were synthesized using cDNA Reverse Transcription Kits (RR092A, Takara bio, Beijing, China) and were determined by the CFX96 Real-Time PCR system (Bio-Rad, Hercules, CA, USA) using SYBR green qPCR master mix (RR820A, Takara bio, Beijing, China) according to the manufacturer’s instructions. Data were analyzed with 2^−ΔΔCt^ value calculation, using β-actin for normalization. The primers for mRNA used in the present study were synthesized at Sangon biotech (Shanghai, China) as *Beclin-1* (F: 5′-GCCTCTGAAACTGGACACGA-3′, R: 5′-CTCCCCGATCAGAGTGAAGC-3′), *Atg5* (F: 5′-CACCCCTGAAATGAGAGTTTTCCA-3′, R: 5′-AAAGTGAGCCTCAACCGCAT-3′), *HMGB1* (F: 5′-ATGACAAGCAGCCCTATGAGAA-3′, R: 5′-CCTTTAGCTCTGTAGGCAGCAA-3′), and *LC3* (F: 5′-GGGACCCTAACCCCATAGGA-3′, R: 5′-GGCACCAGGAACTTGGTCTT-3′). For miRNA, the miDETECT A Track miRNA qRT-PCR Starter Kit was used (C10712-1, RIBOBIO, Guangzhou, China), and data were analyzed with 2^−ΔΔCt^ value calculation, using U6 (miRAN0002-1-100, RIBOBIO, Guangzhou, China) for normalization.

### 2.9. Western Blot

The proteins of the adipose-derived exosomes or right lungs were extracted with RIPA lysis buffer containing protease and phosphatase inhibitors. The lysed samples were incubated on ice for 30 min and centrifuged at 10,000× *g* for 10 min at 4 °C. The lysates were collected, and protein concentrations were measured. Approximately 40 μg of total protein was loaded into each lane for SDS-PAGE. After electrophoresis, the proteins were transferred onto nitrocellulose membranes followed by 5% nonfat dry milk blocking and the primary antibodies incubated overnight at 4 °C. The primary antibodies were CD9, CD63, Alix, HMGB1, LC3, Atg5, Beclin-1, and β-actin. All protein-specific primary antibodies were used at a 1:1000 dilution, and the secondary antibody was used at a 1:20,000 dilution. The blots were developed with the biotin-conjugated secondary antibodies (Cell signaling technology, 7074, Beverly, MA, USA) and visualized by ECL™ (Beyotime technology, P0018A, Shanghai, China). Then, the statistical data analyses of protein expression levels were performed with Quantity One v4.6.6 software.

### 2.10. Nanoparticle Tracking Analysis (NTA)

The adipose-derived exosomes were diluted with 1 × PBS and used directly for NTA detection by PARTICLE METRIX (ZetaVIEW, Munich, Germany), and the diameter and particle concentration of exosomes were visualized by OriginPro 2022 software.

### 2.11. Transmission Electron Microscopy (TEM)

The adipose-derived exosomes suspended in PBS (10 μL) were absorbed onto copper grids for 1 min at room temperature. After excess liquid was removed, samples were stained with methyl cellulose uranyl acetate for 1 min at room temperature. Excess liquid was wicked off the grid using filter paper, and grids were stored at room temperature until imaging.

### 2.12. Statistical Analysis

All statistical analyses were performed in SPSS (26.0, IBM, New York, NY, USA) and visualized by GraphPad (9.0). All data were represented as the mean ± standard deviation (X ± SD). The Shapiro–Wilk test was conducted to evaluate the normality of the data. If the data followed a normal distribution, the differences between/among groups were analyzed with an independent-samples *t* test or one-way analysis of variance (ANOVA); conversely, the Mann–Whitney U test or Kruskal–Wallis test was applied. The correlations were assessed using Spearman’s test. A *p* value < 0.05 was considered statistically significant, and the exact *p* values were labeled for statistically significant data in the figure.

## 3. Results

### 3.1. Characteristics of DIO Mouse Model and Adipose-Derived Exosome

**Figure 1:**
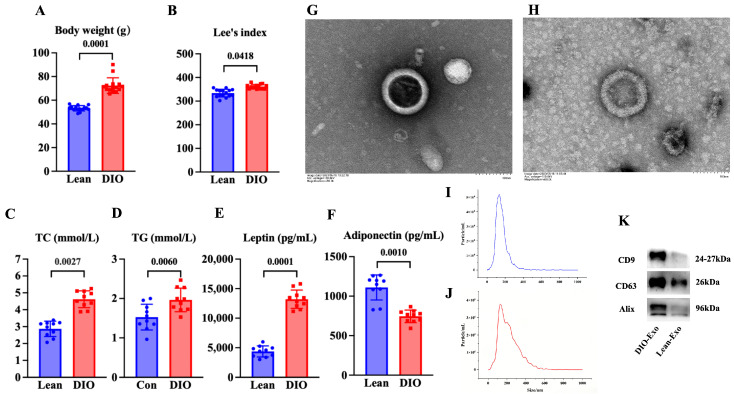
Characteristics of DIO mouse model and adipose-derived exosome. (**A**) Body weight and (**B**) Lee’s index (N = 15), (**C**) TC content and (**D**) TG content (N = 10), and (**E**) leptin level and (**F**) adiponectin level of mice fed with diets after 12 weeks (N = 10). (**G**,**H**) Representative images obtained by TEM of DIO-Exo and Lean-Exo, respectively. (**I**,**J**) Size distributions of DIO-Exo and Lean-Exo, respectively. (**K**) Levels of CD9, CD63, and Alix of DIO-Exo and Lean-Exo.

Compared with the lean mice, the body weight and Lee’s index of the mice in the DIO mice were significantly increased (*p* < 0.05) (Figure 1A,B). The content of serum adiponectin of the DIO mice was significantly decreased in comparison to that in the lean mice (*p* < 0.05) (Figure 1C). The contents of serum leptin, TC, and TG in the DIO mice were significantly increased compared with those in the lean mice (*p* < 0.05) (Figure 1D–F). These results indicated that the DIO mouse model was successfully established by feeding a high-fat diet for 12 weeks.

In TEM, DIO-Exo and Lean-Exo showed the typical bilayer of exosomes (Figure 1G,H). The median sizes of DIO-Exo and Lean-Exo were 140.7 nm and 121.4 nm (Figure 1I,J). Through Western blotting, the DIO-Exo and Lean-Exo were positive for CD9, CD63, and Alix (Figure 1K). These results demonstrated that the exosomes derived from adipose tissue of DIO mice and lean mice were successfully isolated.

### 3.2. Adipose-Derived Exosomes Mitigated Pulmonary Injury of ALI Mouse by Suppressing Autophagy

**Figure 2:**
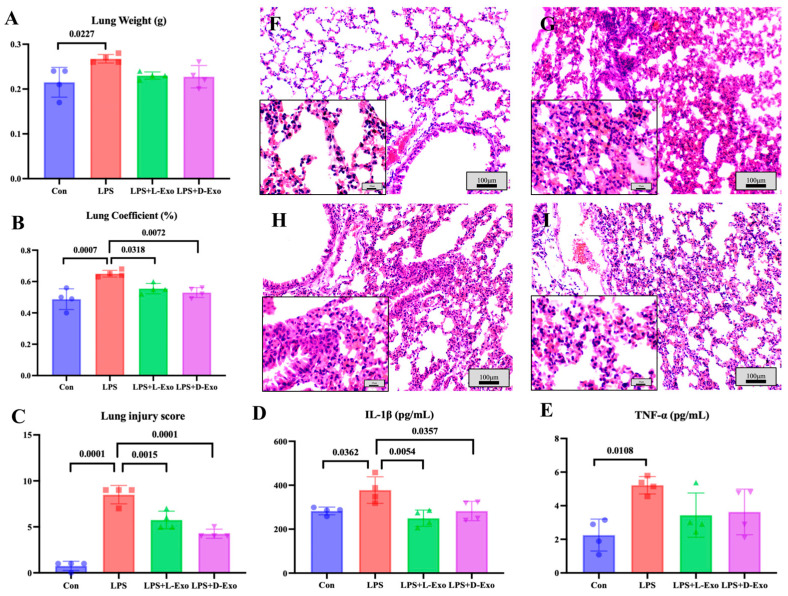
Assessments of lung injury by lung weight, lung coefficient, and histopathological damage. (**A**) Lung weight. (**B**) Lung coefficient. (**C**) Lung injury score. (**D**) Serum IL-1β levels. (**E**) Serum TNF-α levels (N = 4). (**F**–**I**) Represent images of histopathological observation from Con, LPS, LPS + L-Exo, and LPS + D-Exo groups, respectively. H.E. staining. Scale bar: 100 μm or 25 μm.

As the lung weight and lung coefficient could be used to assess the degree of pathological changes, the lung weight and coefficient were determined in the present study. Compared with the Con group, the lung weight and lung coefficient significantly increased in the LPS group (*p* < 0.05). The lung weight of the LPS group was significantly higher than that in the LPS + L-Exo or LPS + D-Exo group (*p* < 0.05), and the lung coefficient of the LPS group was markedly higher than that in the LPS + D-Exo group (*p* < 0.05). And the differences in lung weight and coefficient between the LPS + L-Exo and LPS + D-Exo groups were not statistically significant (*p* > 0.05) (Figure 2A,B).

Histopathological assessments showed that the lung tissue structure of mice in the Con group was essentially normal (Figure 2F), while the alveolar walls in the lung tissues of mice in the LPS group were widely thickened after being treated with LPS by nasal instillation. The alveolar wall thickening was caused by congestion of the alveolar capillaries, edema, and serous exudation (Figure 2G). After being treated with DIO-Exo and Lean-Exo, the pulmonary injuries were relieved, mainly manifest in edema and serous exudation weakening (Figure 2H,I). Histological injury was scored via blind pathological assessment. The LPS group presented the highest lung injury score, which was significantly higher than those of other groups (*p* < 0.05). After we intervened with adipose-derived exosomes, all lung injury scores exhibited a decreasing tendency. Specifically, the LPS + D-Exo group had a significantly lower score in comparison with the LPS group (*p* < 0.05) (Figure 2C). The levels of serum inflammatory factors were consistent with the histological findings. When compared with the Con group, the levels of serum IL-1β and TNF-α were significantly increased in the LPS group (*p* < 0.05). However, these levels showed a decreasing trend after the intervention with adipose-derived exosomes (Figure 2D,E).

**Figure 3:**
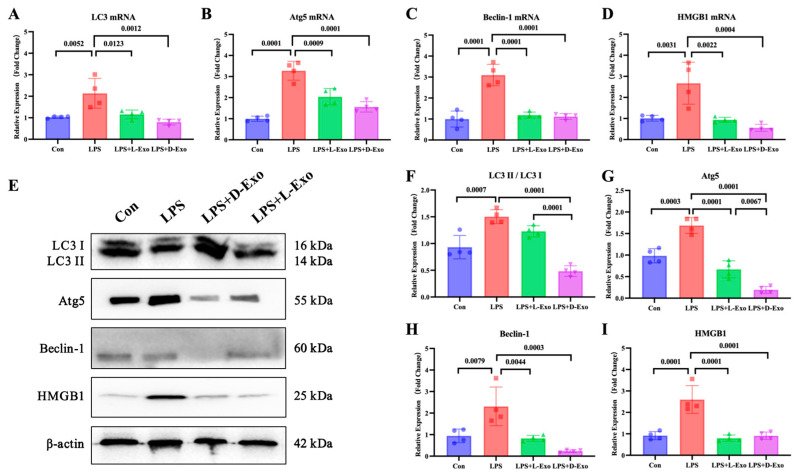
Adipose-derived exosomes mitigated pulmonary injury of ALI mouse by suppressing autophagy. (**A**–**D**) mRNA expression and (**F**–**I**) protein levels of autophagy-related factors in lungs of mice treated with LPS and adipose-derived exosomes (N = 4). (**E**) A representative Western blot depicting protein expression.

To evaluate the pulmonary autophagy, the pulmonary expression levels of autophagy-related factors, including LC3, Atg5, Beclin-1, and HMGB1, were detected. Compared with the Con group, mRNA and protein expression levels of these autophagy-related factors were significantly elevated in the LPS group (*p* < 0.05). Compared with the LPS group, the mRNA and protein levels of autophagy-related factors were markedly decreased in the LPS + L-Exo or LPS + D-Exo group (*p* < 0.05). There were no significant differences in mRNA expression levels of autophagy-related factors between the LPS + L-Exo and LPS + D-Exo groups (*p* > 0.05). Moreover, apart from HMGB1, the protein contents of autophagy-related factors in the LPS + D-Exo group were evidently lower than those in the LPS + L-Exo group (*p* < 0.05) (Figure 3).

### 3.3. MiR-142a-3p Carried by Adipose-Derived Exosomes Alleviated LPS-Induced Lung Injury and Autophagy by Targeting HMGB1

**Figure 4:**
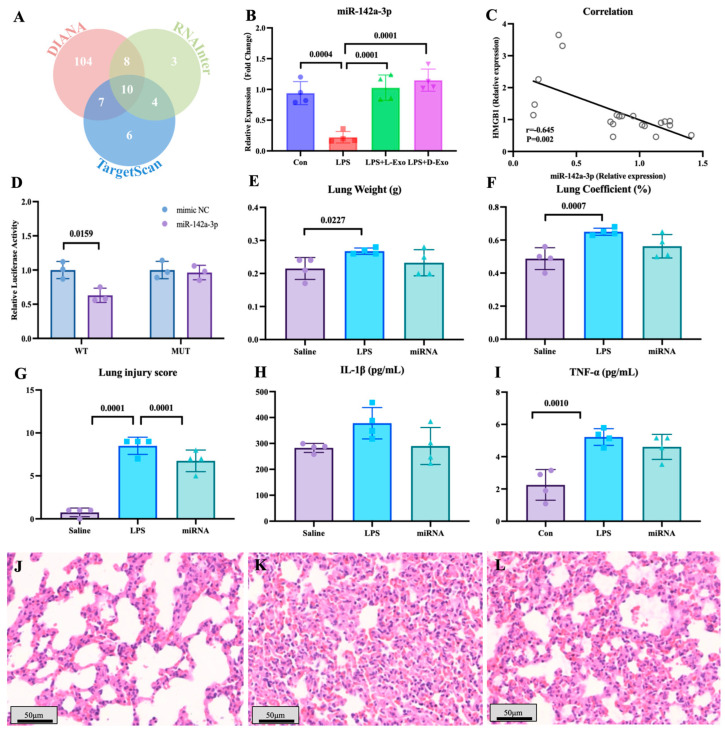
miR-142a-3p carried by adipose-derived exosome targets to HMGB1 alleviated LPS-induced lung injury. (**A**) Bioinformatic analysis of miRNAs targeting HMGB1. (**B**) Levels of pulmonary miR-142a-3p in the mice treated with LPS and adipose-derived exosomes (N = 4). (**C**) Correlation between pulmonary miR-142a-3p and HMGB1. (Note: Each circle represents an observation point corresponding to an individual subject, where the X-axis and Y-axis represent the expression levels of miR-142a-3p and *HMGB1*, respectively. Same for Figure 5J) (**D**) Relative luciferase activity of 293T cell transfected with reporters and miR-142a-3p (N = 3). (**E**) Lung weight. (**F**) Lung coefficient. (**G**) Lung injury score. (**H**) Serum IL-1β levels. (**I**) Serum TNF-α levels. (N = 4) (**J**–**L**) Representative images of histopathological observation from saline, LPS, and miRNA groups, respectively. H.E. staining. Scale bar: 50 μm.

To explore the functional components of adipose-derived exosomes, the miRNAs carried by adipose-derived exosomes and potentially targeted to HMGB1 mRNA were screened by bioinformatics analysis (Figure 4A). Of miRNAs carried by adipose-derived exosomes, the level of pulmonary miR-142a-3p in the LPS group significantly declined in comparison to the saline group, and the level of miR-142a-3p in the LPS + L-Exo or LPS + D-Exo group significantly increased when compared with the LPS group (*p* < 0.05) (Figure 4B). Furthermore, the level of pulmonary miR-142a-3p was negatively correlated with the level of *HMGB1* (*p* < 0.05) (Figure 4C). Moreover, the data of the dual-luciferase reporter assay showed that miR-142a-3p significantly down-regulated the relative luciferase activity of the reporter containing the wild type of *HMGB1* (*p* < 0.05), while not affecting the relative luciferase activity of the reporter containing mutant *HMGB1* (*p* > 0.05) (Figure 4D).

The lung weight in the LPS group was significantly higher than that in the saline group (*p* < 0.05), and the lung coefficient in the LPS group was markedly higher than that in the saline and miRNA groups (*p* < 0.05) (Figure 4E,F). As shown in Figure 4G–I, LPS caused serious alveolar wall thickening with congestion, and the miRNA group widened the area of the pulmonary ventilation region. The lung injury score indicated that subsequent to the intervention with miR-142a-3p, the histopathology score significantly decreased in comparison with the LPS group (*p* < 0.05) (Figure 4G). The levels of serum IL-1β and TNF-α both exhibited a decreasing tendency following the intervention with miR-142a-3p (Figure 4H,I).

**Figure 5:**
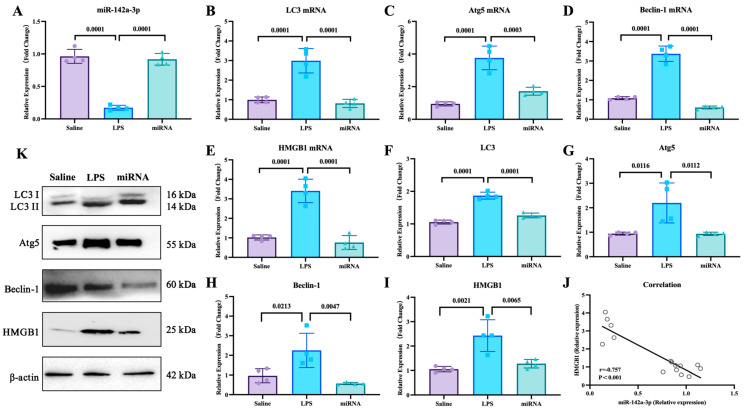
Expression levels of miR-142a-3p and autophagy-related factors in lungs of mice treated with LPS and miR-142a-3p. (**A**) Levels of pulmonary miR-142a-3p. (**B**–**E**) mRNA expression levels and (**F**–**I**) protein contents of pulmonary autophagy-related molecules. (N = 4) (**J**) Correlation of miR-142a-3p and HMGB1 level. (**K**) Representative images of protein levels of pulmonary autophagy-related molecules determined by Western blot.

With the mouse model, the effects of miR-142a-3p in ALI were investigated. As shown in Figure 5, LPS administration down-regulated the expression of pulmonary miR-142a-3p (*p* < 0.05), while miR-142a-3p agomir significantly increased pulmonary levels of miR-142a-3p in the mice treated with LPS (*p* < 0.05). Compared with the saline group, the mRNA and protein expression levels of autophagy-related factors in the lungs were significantly up-regulated (*p* < 0.05). And the mRNA and protein levels of autophagy-related factors in the miRNA group were markedly lower than those in the LPS group (*p* < 0.05). Moreover, the pulmonary expression of HMGB1 was negatively correlated with the levels of miR-142a-3p (*p* < 0.05).

**Figure 6:**
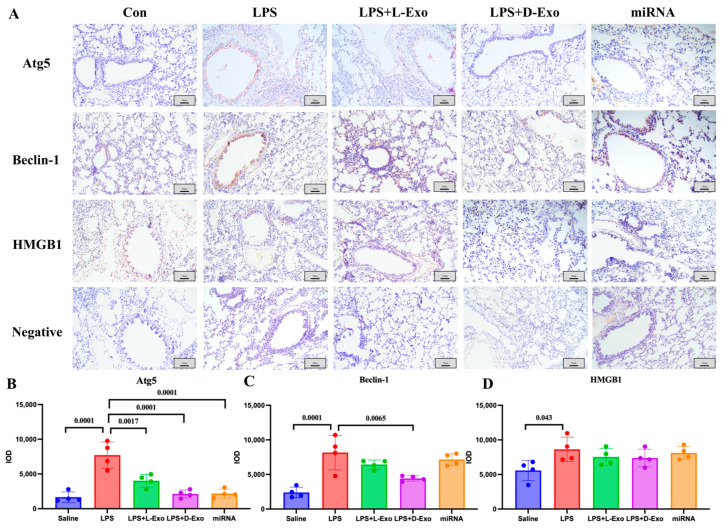
The locations of Atg5, Beclin-1, and HMGB1 in lungs. (**A**) Distribution of HMGB1, Atg5, and Beclin-1 in lungs of mice in each group determined using immunohistochemical staining. Scale bar: 100 μm. (**B**–**D**) Semiquantitative analysis of positive protein deposition using Integrated Optical Density (IOD) measurement. (N = 4).

The locations of Atg5, Beclin-1, and HMGB1 in the lungs were analyzed using an immunohistochemical assay. In the Con or saline group, the immunohistochemical staining of Atg5, Beclin-1, and HMGB1 showed a relatively weak positive signal. In the LPS group, the positive protein of Atg5 was mainly expressed in the cytoplasm of alveolar epithelial cells, Beclin-1 was expressed in the cytoplasm of the epithelial cells of the bronchioles, where it mainly presented in the free surface, along with a small amount in the cytoplasm of alveolar epithelial cells, while HMGB1 was concentrated in the cytoplasm and cell nucleus in the epithelial basal-layer cells of the pulmonary airways, bronchioles, and terminal bronchioles. In the LPS + L-Exo and LPS + D-Exo groups, the expression of Atg5, Beclin-1, and HMGB1 was relatively lower than that of the ALI group, especially in the LPS + D-Exo group, whereas there was no significant difference. Moreover, in the miRNA group, the expression levels of Atg5 and Beclin-1 were relatively decreased (Figure 6).

## 4. Discussion

Acute lung injury (ALI) is caused by the impairment of alveolar epithelial cells and capillary endothelial cells due to various direct and indirect injury factors, leading to diffuse pulmonary interstitial and alveolar edema and acute hypoxic respiratory insufficiency. Pathophysiologically, it is characterized by a reduced lung volume, decreased lung compliance, and ventilation/perfusion mismatch in terms of pathophysiology. The present study demonstrated that LPS administration caused histopathological damage of the lungs, including clear thickening of the alveolar walls, hemorrhage and infiltration of immune cells, and infiltration, consistent with the previous research on LPS-induced ALI [29]. And adipose-derived exosomes (ADEs) from either lean or DIO mice exhibited protective effects against ALI, in accordance with prior studies [10,30]. Wang et al. reported that ADEs protected mice from LPS-induced ALI by attenuating pulmonary inflammation and the cell cycle [10], and Xia et al. found that adipose-derived mesenchymal stem cell-derived exosomes improved the homeostasis of macrophages by transferring mitochondrial components and alleviated LPS-induced ALI [30]. Our data indicated that the protective effect of ADEs was created by inhibiting pulmonary autophagy mediated by HMGB1 in the setting of ALI. It has been reported that the exosomes from multiple resources could be taken up by pulmonary alveolar epithelial cells and alveolar macrophages and regulate autophagy in ALI [31,32,33,34,35]. In the previous research studies, promotion of autophagy by exosomes in alveolar epithelial cells attenuated ALI of animal models [31,32], and either inhibition or promotion of autophagy in the alveolar macrophages by exosomes significantly relieved ALI [33,34,35]. And autophagy is triggered at the initial stage of sepsis development, but autophagic flux decreases with the progression of sepsis [36]. Based on these data, we speculated that ADEs suppressed the excessive autophagy of alveolar macrophages or alveolar epithelial cells and mitigated ALI at an early stage, and the target cells of ADEs warrant further investigation.

To explore the functional components of adipose-derived exosomes on anti-autophagy in ALI, we focused on HMGB1 and miRNA carried by exosomes. HMGB1, a damage-associated molecular pattern widely distributed in lungs, plays a crucial role in controlling autophagy [37]. It has been demonstrated that HMGB1-driven autophagy was implicated in ALI [38], and HMGB1 was a potential therapeutic target for ALI [39]. MicroRNAs (miRNAs), a class of small, non-coding RNA molecules, primarily target the silencing process of downstream genes. Thomou et al. reported that adipose tissue is the main source of circulating exosomal miRNAs [40]. Several research studies have shown that various miRNAs regulate autophagy by modulating gene expression, including miR-377-3p/RPTOR [31], miR-100/mTOR [32], miR-451/TSC1 [33], miR-384-5p/Beclin-1 [34], and miR-223-3p/STK39 [35]. In the present study, in the dual-luciferase reporter assay, miR-142a-3p could specifically bind to *HMGB1* mRNA, as previously reported in the mouse liver [41]. And we firstly found that the pulmonary *HMGB1* level was negatively correlated with the expression of pulmonary miR-142a-3p, and up-regulation of pulmonary miR-142a-3p suppressed pulmonary autophagy and alleviated lung injury in the ALI model. As reported by Yang et al., miR-142a-3p significantly reduced the secretion of pro-inflammatory cytokines by inhibiting the TLR4/TAB2/NF-κB pathway in RAW264.7 cells [42]. Therefore, the protective role of miR-142a-3p may be arise in independent ways, including anti-autophagy and anti-inflammation.

And, in the present study, the effect of ADEs and miR-142a-3p against ALI was investigated at a single time point (12 h post-ALI) when the severe lung injuries were observed [43,44]. During the development of ALI, pulmonary autophagy and injury undergo dynamic alterations [36,45]. These data indicated that the impact of autophagy varies across different stages of ALI, and the role of autophagy regulated by ADEs at multiple time points during ALI warrants further elucidation.

Moreover, exosomes from the lean or DIO mice showed comparable regulation of autophagy-related mRNAs, not proteins, which could result from the similar miRNA profile regulating autophagy in the exosomes. Wang et al. found there were 40 differentially expressed miRNAs between the L-Exo and D-Exo [10], of which the miRNAs’ degrees of potential to regulate the autophagy-related mRNAs, including miR-377-3p, miR-384-5p, miR-451, miR-142-3p, miR-223-3p, and miR-100, were not significantly different between the exosomes from the lean and DIO mice. And the protein levels of multiple molecules associated with autophagy (LC3 II/LC3 I, Atg5, and Beclin-1) in the LPS-D-Exo mice were significantly lower than those in the LPS-L-Exo mice, which might be the result of other effective components carried by adipose-derived exosomes, like proteins, lncRNAs, or circRNAs.

However, there are four limitations of this study. 1. The effects of adipose-derived exosomes and miR-142a-5p were investigated by in vivo experiments with small animal numbers, and BALF characterization was not used to assess the lung injury of ALI mice. 2. The specific target cells of adipose-derived exosomes and miR-142a-3p remain ambiguous, and the mechanism of adipose-derived exosomes and miR-142a-3p mitigating ALI by targeting HMGB1 and autophagy should be verified by in vitro experiments using pulmonary alveolar epithelial cells and alveolar macrophages. 3. The pulmonary autophagy affecting adipose-derived exosomes and miR-142a-3p was detected at one timepoint (12 h post-ALI), and autophagic flux could be further investigated to illustrate dynamic changes in autophagy during ALI. 4. The contribution of miR-142a-3p to the protective effects of adipose-derived exosomes against ALI is unclear; loss-of-function experiments would be helpful to demonstrate the role of miR-142a-3p.

## 5. Conclusions

Adipose-derived exosomes inhibited pulmonary HMGB1-driven autophagy and alleviated pulmonary injuries in vivo at 12 h post-ALI caused by LPS. We found that miR-142a-3p might be the effective factor, which should be a potential target of new strategies for treating ALI.

## Data Availability

Data is contained within the article. Further inquiries can be directed to the corresponding author.
